# Antiplasmodial and trypanocidal activity of violacein and deoxyviolacein produced from synthetic operons

**DOI:** 10.1186/s12896-018-0428-z

**Published:** 2018-04-11

**Authors:** Elizabeth Bilsland, Tatyana A. Tavella, Renata Krogh, Jamie E. Stokes, Annabelle Roberts, James Ajioka, David R. Spring, Adriano D. Andricopulo, Fabio T. M. Costa, Stephen G. Oliver

**Affiliations:** 1 Cambridge Systems Biology Centre and Department of Biochemistry, University of Cambridge, Cambridge, UK; 2 Department of Structural and Functional Biology, Institute of Biology, UNICAMP, Campinas, SP Brazil; 3 Laboratory of Tropical Diseases – Prof. Dr. Luiz Jacintho da Silva - Department of Genetics, Evolution, Microbiology and Immunology, University of Campinas, Campinas, SP Brazil; 4 Laboratory of Medicinal and Computational Chemistry, University of São Paulo, São Carlos, SP Brazil; 5 Department of Chemistry, University of Cambridge, Cambridge, UK; 6 Department of Pathology, University of Cambridge, Cambridge, UK

**Keywords:** Violacein, Deoxyviolacein, *Plasmodium falciparum*, *Trypanosoma cruzi*, Synthetic operon, Antiparasitic, *Escherichia coli*

## Abstract

**Background:**

Violacein is a deep violet compound that is produced by a number of bacterial species. It is synthesized from tryptophan by a pathway that involves the sequential action of 5 different enzymes (encoded by genes *vio*A to *vio*E). Violacein has antibacterial, antiparasitic, and antiviral activities, and also has the potential of inducing apoptosis in certain cancer cells.

**Results:**

Here, we describe the construction of a series of plasmids harboring the complete or partial violacein biosynthesis operon and their use to enable production of violacein and deoxyviolacein in *E.coli*. We performed *in vitro* assays to determine the biological activity of these compounds against *Plasmodium*, *Trypanosoma,* and mammalian cells. We found that, while deoxyviolacein has a lower activity against parasites than violacein, its toxicity to mammalian cells is insignificant compared to that of violacein.

**Conclusions:**

We constructed *E. coli* strains capable of producing biologically active violacein and related compounds, and propose that deoxyviolacein might be a useful starting compound for the development of antiparasite drugs.

**Electronic supplementary material:**

The online version of this article (10.1186/s12896-018-0428-z) contains supplementary material, which is available to authorized users.

## Background

Violacein is a violet indolocarbazole pigment that is produced by bacteria such as *Chromobacterium violaceum*, which are commonly found in water and soil throughout the world [1–6]. Violacein has antipyretic [7, 8], ulcer-protective [8], antibacterial [9–11], antifungal [3, 12], trypanocidal [13, 14], antileishmanial [15], antinematode [16], and antiviral [17] activities. It also has the potential of inducing apoptosis in certain cancer cells [2, 18]. Violacein kills wild-type and drug-resistant strains of the malaria parasite, *Plasmodium falciparum* and is therapeutically against malaria in mice [19]. These characteristics suggest that violacein has considerable research potential and may represent a chemical scaffold for the developemt of clinically useful drugs.

Commercially, violacein is usually isolated from *Chromobacterium* [20–22] or *Janthinobacterium* [23, 24]; however, this process is costly and there are reports of rare but deadly infections caused by these bacteria [25–28]. Hence, there has been considerable interest in the development of safe, and efficient routes to the biosynthesis of this compound [1, 29–34].

**Fig. 1:**
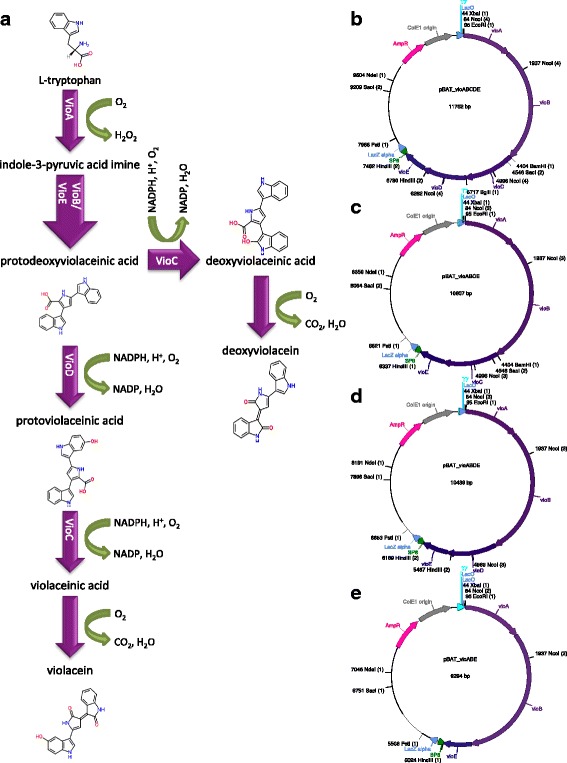
Violacein biosynthetic pathway (**a**) and plasmid maps of the complete (**b**) and partial (**c, d** and **e**) operons for violacein biosynthesis

The violacein biosynthetic pathway from L-tryptophan (Fig. 1) requires the expression of five genes: *vioA, vioB, vioC, vioD*, and *vioE* [35–39]. It should be noted that VioC enzyme is involved in both the production of deoxyviolacien from protodeoxyviolaceinic acid and in the generation of violacein from protoviolaceinic acid. Several studies have shown that transforming and expressing a complete metabolic pathway into a different bacterial host may lead to improved production of violacein [40–43]. For example, Rodrigues and co-workers [32–34] have successfully engineered *Escherichia coli* to produce high yields of violacein and the side-product deoxyviolacein. This was accomplished by cloning the complete *vioABCDE* and the partial *vioABCE* operons (respectively) from *C. violaceum* into pBADMycHisB, which allows the induction of the operon by L-arabinose [32, 34]. These authors also metabolically engineered the host’s tryptophan production to maximize the yield of violacein and deoxyviolacein [32, 34, 42]. More recently Jones and co-workers [44] and Xu and co-workers [45] utilized violacein biosynthesis as a model for metabolic pathway balancing and optimization. Employing different approaches, they fine-tuned the expression of violacein-producing enzymes, leading to an improvement in the production of the compound by up to 30-fold when compared to previously reported work.

We have generated a synthetic operon containing the coding sequences of each of the five genes required for violacein biosynthesis, with a codon-usage optimized for *E. coli* (http://parts.igem.org/Part:BBa_K274002). We also constructed strains lacking *vioD*, to promote the accumulation of deoxyviolacein. A similar approach was employed by Rodrigues and co-workers for the production of high yields of violacein and deoxyviolacein [32–34]. We produced and purified violacein and deoxyviolacein, and characterized their toxicity and antiplasmodial activity in wild-type and drug-resistant *Plasmodium falciparum* strains; we also determined their activity against *Trypanosoma cruzi*.

## Methods

### Bacterial strains and plasmids

We constructed plasmids expressing violacein and deoxyviolacein by sub-cloning the synthetic violacein operon (Part: BBa_K274002) designed by Shuna Gould for the iGEM09_Cambridge project (http://parts.igem.org/Part:BBa_K274002). The synthetic violacein operon is comprised of the 5 coding sequences specifying violacein pathway enzymes (*vioA, vioB, vioC*, *vioD* and *vioE*), each preceded by a ribosome-binding site. The operon was designed with a *Bam*HI site in the space between *vioB* and *vioC* open reading-frames (ORFs), a *Bgl*II site between the *vioC* and *vioD* ORFs, and a *Bcl*I site between the *vioD* and *vioE* reading-frames. Since cleavage of the *Bam*HI, *Bgl*II, and *Bcl*I sites generate compatible cohesive ends, this facilitated the construction of three different operons: *vioABCE*; *vioABDE*; *vioABE*. The synthetic operons are flanked by *Eco*RI and *Pst*I restriction endonuclease sites, enabling the use of these two enzymes to readily subclone the entire (*vioABCDE*) and partial operons (*vioABCE*, *vioABDE*, *vioABE*) into the *Eco*RI and *Nsi*I sites of pBAT4 (Fig. 1).

### Production and purification of violacein and deoxyviolacein

We transformed *E. coli* BL21(DE3) (New England Biolabs) cells with plasmids expressing the synthetic *vioABCDE* (for production of both violacein and deoxyviolaein) or *vioABCE* (for production of deoxyviolacein alone) operons. The leaky expression from the T7 promoter in these plasmids was enough to allow sufficient synthesis of the enzymes in the violacein biosynthetic pathway.

We picked individual colonies, inoculated 50 mL cultures in 2× YT (16 g/L Tryptone, 10 g/L yeast extract, 5 g/L sodium chloride) with 100 mg/L ampicillin, and incubated for 16 h at 37 °C. These pre-cultures were inoculated into 20 L of 2xYT supplemented with 100 mg/L of ampicillin and 100 mg/L of L-tryptophan in a Sartorius Biostat Cplus fermenter. Cultures were grown for 5 h at 37 °C, with agitation (400 rpm) and air influx of 4 L/min. The temperature was then reduced to 20 °C (to avoid excessive growth and foaming overnight) and the cultures were incubated for a further 16 h. Cells were harvested by centrifugation. Violacein and deoxyviolacein were extracted by resuspending the bacterial pellets in 500 mL of 90% *v*/v acetone. Cell suspensions in acetone were filtered to produce crude violacein and deoxyviolacein extracts.

### Violacein and deoxyviolacein purification

#### Deoxyviolacein

The acetone cell extract was evaporated to dryness. The crude residue was suspended in acetone and dry-loaded onto silica gel (SiO_2_). Deoxyviolacein was purified by column chromatography on silica gel (SiO_2_), first washing with petroleum ether (boiling point = 40–60 °C) and eluting with a 1:1 solution of ethyl acetate and petroleum ether. Deoxyviolacein was obtained as a purple solid and was analytically pure (100%). The identity and purity of deoxyviolacein were confirmed by ^1^H NMR (Additional file 1: Figure S1). We calculated the purity by integrating related peaks and comparing the areas. No other analysis was run because the data is consistent with that from the literature [46]. The apparent purity of deoxyviolacein allowed its quantitation and that of violacein (see below). Minor contamination by inorganic compounds cannot be excluded but these would need to be soluble in acetone.

#### Violacein

The acetone cell extract was evaporated to dryness. The crude residue was suspended in acetone with sonication and dry-loaded onto silica gel (SiO_2_). Violacein was purified by column chromatography on silica gel (SiO_2_), first washing with petroleum ether (boiling point = 40–60 °C) and eluting with 4:6, 1:1, and 6:4 solutions of ethyl acetate and petroleum ether (boiling point = 40–60 °C). Violacein was obtained as a crude mixture with approximately 12% deoxyviolacein (estimated from ^1^H NMR). The identity of violacein was confirmed by ^1^H NMR (Additional file 1: Figure S1) and was consistent with the literature [46].

### *Plasmodium falciparum* drug sensitivity assays

We cultivated *Plasmodium falciparum* 3D7 and W2 strains in complete RPMI (RPMI 1640, Sigma, USA), supplemented with 10% plasma (AB^−^) and 2% Haematocrit (O^+^). *Plasmodium* cultures were synchronized twice with sorbitol, and drug sensitivity tests were performed on cultures enriched for ring-stage parasites. The parasitemia of the cultures was adjusted to 1% and drug sensitivity screens were performed in 96-well plates, with the following drug concentrations: for violacein and chloroquine [47] 8 concentrations of a 2× serial dilution, starting with 5 μM were employed; for deoxyviolacein, the same number of serial dilutions were tested with a starting concentration of 50 μM. All experiments were performed in triplicate and included solvent controls as well as untreated erythrocytes. After 48 h of incubation at 37 °C, cultures were labeled with SYBR® Green and analyzed by flow cytometry. IC50s were calculated using GraphPad Prism version 5.01.

### *Trypanosoma cruzi* drug sensitivity assays

*In vitro* drug sensitivity assays on *Trypanosoma cruzi* were performed as described by Ferreira [48]. Briefly, we performed the assays using *T. cruzi* strain Tulahuen (parasites engineered to express *E. coli* β-galactosidase, *lacZ* [49], that catalyzes a colorimetric reaction when biologically active). Trypomastigotes were grown on monolayers of human fibroblasts, and epimastigotes were grown in liver infusion tryptone with 10% fetal calf serum, penicillin and streptomycin (to prevent contamination). Cultures assayed for β-galactosidase activity were grown in RPMI 1640 medium without phenol red plus 10% fetal calf serum, penicillin, and streptomycin.

Drug-sensitivity assays were performed in 96-well tissue culture plates (Becton Dickinson). Human fibroblasts were seeded at 2 × 10^3^ per well in 80 μL volumes (RPMI 1640 without phenol red) and incubated overnight. The next day, β–galactosidase-expressing trypomastigotes were added at 1 × 10^4^ per well in 20 μL of RPMI 1640 without phenol red. After 24 h, violacein or deoxyviolacein (10 mM stocks in DMSO) were added to the cultures in serial dilutions in 50 μL volumes (RPMI 1640 without phenol red). Each dilution was tested in triplicate. After 72 h of incubation, the plates were inspected under an inverted microscope to check the growth of the controls and sterility. Then, 50 μL of the substrate containing chlorophenol red-β-D-galactopyranoside (CPRG) and Nonidet P-40 (0.1% final concentration) was added to all wells. β–galactosidase activity led to a change in the color of the medium from yellow to red, a change that was quantified through measuring the absorbance at 570 nm in an automated plate reader [48]. Wells containing violacein and deoxyviolacein (without phenol red) were used to normalize for the purple color of the compounds. Data were transferred into Sigma Plot to determine IC_50_ values. The drug benzimidazole was used as a positive control and untreated parasite cultures were used as negative control for these assays.

### Cytotoxicity of violacein and deoxyviolacein

The cytotoxicity of the compounds used in this work was evaluated using an MTT [3-(4,5-dimethylthiazol-2-yl)-2,5-diphenyltetrazolium bromide assay. MTT is a yellow tetrazole compound that is reduced to a purple formazan in the mitochondria of living cells. Hence, the proportion of living eukaryotic cells in a given culture can be quantified by monitoring its absorbance at 590 nm.

We cultured HepG2 (human hepatoma) and COS-7 (kidney from African Green Monkey) cell lines in the presence of different concentrations of the test compounds, to evaluate their cytotoxicity. We incubated 10^4^ cells in 200 μL cultures (96-well plates) in 5% *v*/v CO_2_ at 37 °C, in RPMI with Gentamicin (40 mg/L), and 10% of heat-inactivated fetal calf serum (FCS). The final concentrations of test compounds were: violacein (10 μM, 5 μM, 2.5 μM, 1.25 μM and 0.62 μM) and deoxyviolacein (200 μM, 100 μM, 50 μM, 25 μM and 12.5 μM).

After 48 of incubation, we added 15 μL of 5 mg/L MTT and incubated for 4 h in 5% v/v CO_2_ at 37 °C. The plate was then centrifuged at 1500 rpm for 5 min, the supernatant discarded, the cells washed with PBS, and resuspended in 50 μL of isopropanol. Wells containing violacein and deoxyviolacein (without MTT) were used to normalize for the purple color and precipitation of the compounds.

We cultured HepG2 (human hepatoma) and CHO-745 (Chinese hamster ovary) cell lines in the presence of different concentrations of the test compounds, to evaluate their morphology. We incubated 10^4^ cells in 200 μL cultures (96-well plates) in 5% v/v CO_2_ at 37 °C, in RPMI with Gentamicin (40 mg/L), and 10% of heat-inactivated fetal calf serum (FCS). The final concentrations of test compounds were: violacein (0 μM, 0.8 μM, 4 μM, 20 μM and 100 μM) and deoxyviolacein (0 μM, 0.8 μM, 4 μM, 20 μM and 100 μM).

## Results

### Activity of purified violacein and deoxyviolacein against *T. cruzi*

We evaluated the biological activity of violacein and deoxyviolacein produced in our recombinant *E. coli* strains expressing the complete or partial synthetic violacein operon, and found that violacein could efficiently inhibit the trypanosomatids, with an IC_50_ of 1.51 μM ± 0.4, whereas the IC_50_ for deoxyviolacein was above 50 uM. As a comparison, the IC_50_ determined in the same experiment for the anti *T. cruzi* drug benzimidazole (N-benzyl-2-(2-nitro-1H- imidazol-1-yl)acetamide) was 3.07 μM ±0.6. The IC_50_ values of the compounds against the *T. cruzi* Tulahuen strain in the *in vitro* assay represent the means of at least three individual experiments.

### Antiplasmodial activity of purified violacein and deoxyviolacein

**Fig. 2:**
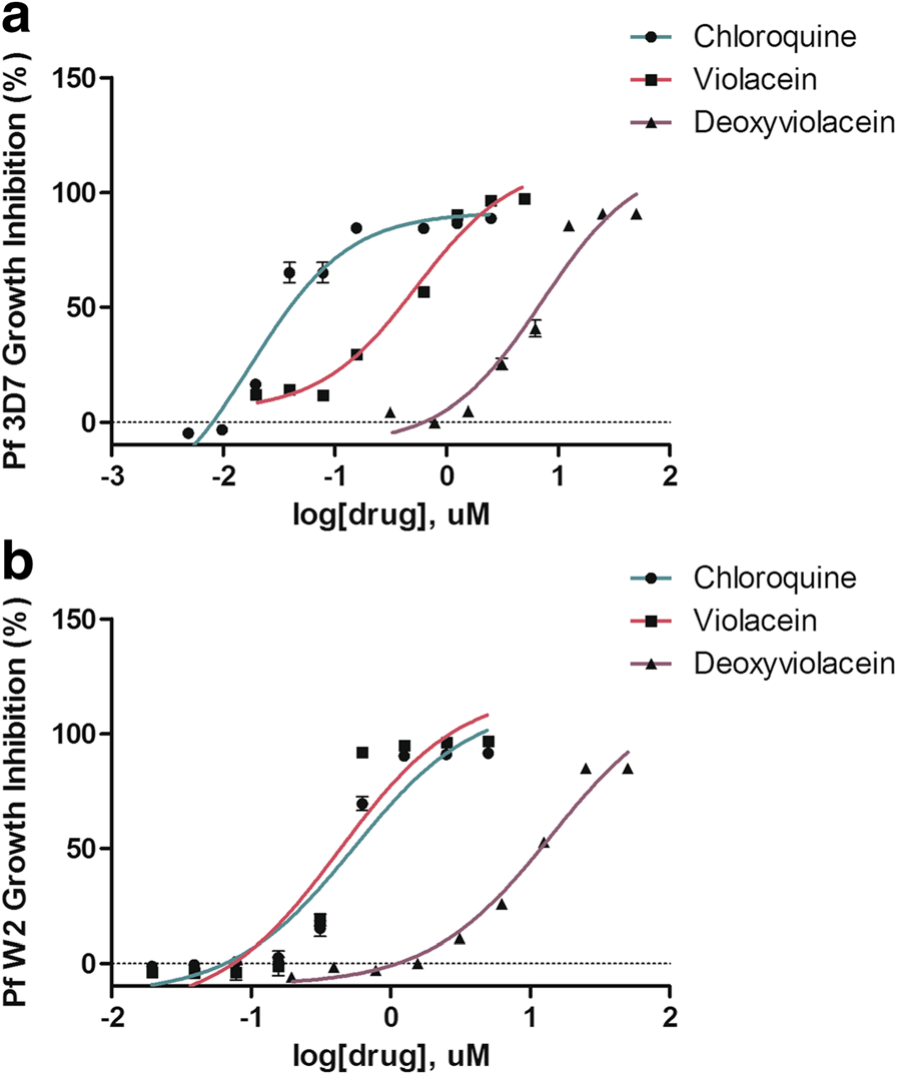
*Plasmodium falciparum* growth inhibition assays. **a** IC50 3D7- Violacein: 0.54 μM ±0.08; Deoxyviolacein: 11 μM ±0.1; Chloroquine: 18 nM ±0.1. **b** IC50 W2- Violacein: 0.42 μM ±0.14; Deoxyviolacein: 14.04 μM ±0.07; Chloroquine: 0.54 μM ±0.11

Initially, we tested higher concentrations (> 5 μM) of violacein in a *Plasmodium* sensitivity test, and noted that samples treated with 10 or 50 μM of violacein could not be analyzed through flow cytometry as all erythrocytes ruptured in the presence of those doses of the compound. Violacein was active against both chloroquine-sensitive and chloroquine-resistant *Plasmodium* strains (IC_50_ ~ 0.4 μM against 3D7 and ~ 0.5 μM against W2 parasites). Deoxyviolacein, on the other hand, showed a mild activity against *Plasmodium* strains when compared with violacein (IC_50_ ~ 11 μM against 3D7 and ~ 14 μM against W2 parasites). The chloroquine control curve performed in both strains showed IC_50_ values comparable with those described in the literature (Fig. 2).

### Cytotoxicity of violacein and deoxyviolacein

When performing drug sensitivity assays on erythrocytes infected with *P.falciparum*, we noticed that healthy and infected erythrocytes ruptured when treated with 10 μM or more of violacein. In contrast, erythrocytes treated with 50 μM deoxyviolacein did not show any obvious morphological changes compared to the untreated cells. We evaluated the morphological changes in HepG2 and CHO-745 cells upon exposure to violacein and deoxyviolacein, but once more detected no morphological changes to cells treated with 20 μM deoxyviolacein and minor changes to cells treated with 100 μM deoxyviolacein (Additional file 2: Figures. S2 and S3).

**Fig. 3:**
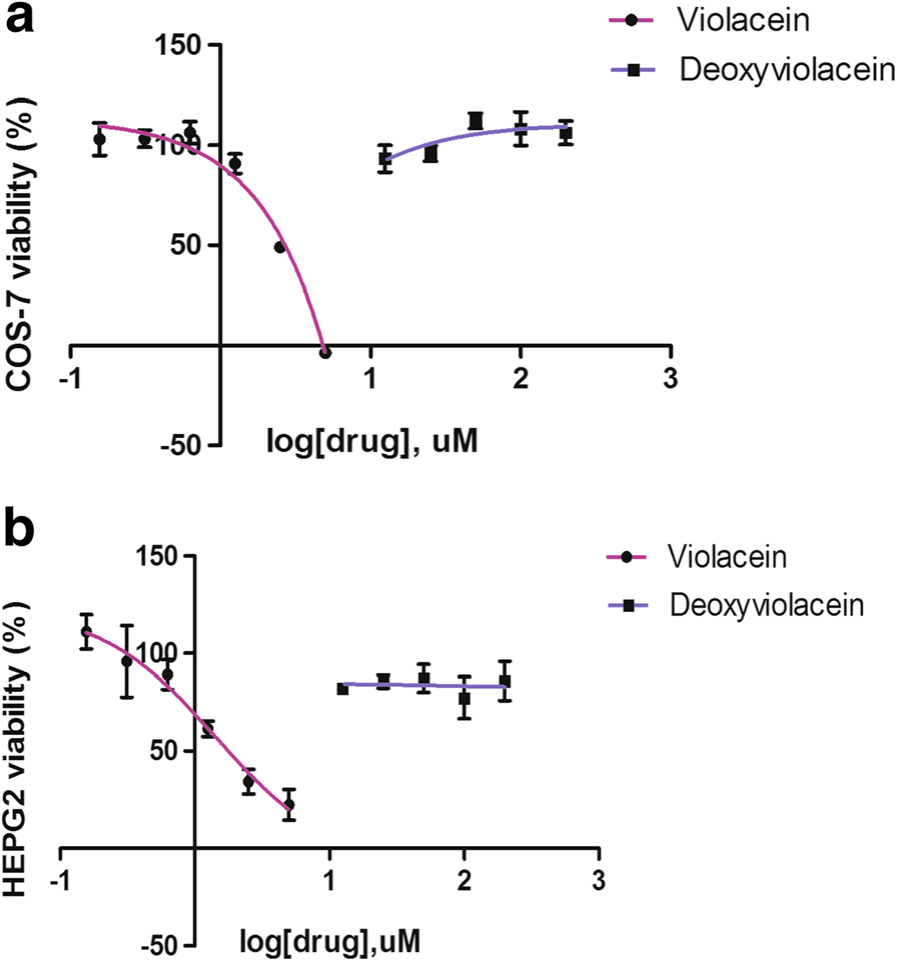
Cytotoxicity of violacein and deoxyviolacein. MTT assay used to investigate the cytotoxicity of violacein and deoxyviolacein to (**a**) COS-7 and (**b**) HepG2 cells, indicated that deoxyviolacein shows no measurable toxicity to mammalian cells, whereas the IC_50_ for violacein is approximately 2.5 μM and 1.4 μM for COS-7 and HepG2 cells, respectively

We performed viability assays to investigate the toxicity of violacein and deoxyviolacein to COS-7 and HepG2 cell lines. Our experiments confirmed the cytoxicity of violacein against both cell lines (IC_50_ of ~ 2.5 μM against COS-7 and ~ 1.4 μM against HepG2), with a stronger effect on the tumor cell line. In contrast, deoxyviolacein showed low toxicity against mammalian cell lines, as cells were able to grow well even in the presence of concentrations of deoxyviolacein 20 times higher then its IC50 in *Plasmodium* strains **(**Fig. 3**).** We were unable to test higher deoxyviolacein concentrations since it precipitated under our experimental conditions.

## Discussion

We have constructed *E. coli* strains producing either a mixture of violacein or dexyviolacein alone, using enzymes encoded by synthetic operons. The biological activity of these compounds against *Trypanosoma*, *Plasmodium* and mammalian cells was assessed. The synthesis of these pigment compounds was easily monitored by their color and it was possible to observe, for example, that pigment formation was greatly enhanced by intense aeration.

We purified deoxyviolacein using the conditions described in the Methods section, achieving close to 100% purity in just a few purification steps. Violacein, however, was contaminated with approximately 12% deoxyviolacein. Hence, in all experiments where we describe the biological effects of violacein, we had some deoxyviolacein as a contaminant. As the biological activity of deoxyviolacein was consistently lower than that of violacein, we inferred that this contamination would not interfere with the interpretation of the results.

We performed *in vitro* assays to investigate the antiplasmodial activity of violacein and deoxyviolacein, using both 3D7 (wild-type) and W2 (chloroquine-resistant) *Plasmodium falciparum* strains. We found that the IC_50_ of violacein was *ca.* 0.5 μM, whereas that of deoxyviolacein was *ca.* 10 μM. The 3D7 and W2 strains were equally sensitive to violacein and deoxyviolacein, whilst 3D7 was around 30 times more sensitive to chloroquine (IC_50_ ~ 20 nM) than W2 (IC_50_ ~ 0.5uM). Hence, the mechanism conferring resistance to chloroquine in W2 lines did not affect their sensitivity to violacein or deoxyviolacein.

When treating *Plasmodium*-infected erythrocytes with violacein, we found that higher concentrations of the pigment caused the complete rupture of the red blood cells. Hence, we decided to investigate the cytotoxicity of violacein and deoxyviolacein. In spite of their very similar structure, violacein was very toxic to mammalian cells, whereas deoxyviolacein showed selective toxicity against *Plasmodium* (the parasite was at least 20× more sensitive to this compound than were the mammalian cells) than violacein (the parasite showing only *ca.* 5× greater sensitivity to this compound than did mammalian cells). The IC_50_ of violacein produced from our synthetic operon is about ~ 1.4 μM for HepG2 and ~ 2.5 μM for COS-7 cell lines, in agreement with published results, indicating a degree of specificity of the compound against cancer cells. On the other hand, the HepG2 cell line showed no significant viability loss when treated with deoxyviolacein concentrations close to 200 μM. When treating the *Trypanosoma cruzi* Tulahuen strain with violacein and deoxyviolacein, we also observed much higher biological activity with violacein (IC_50_ of 1.51 μM ± 0.4) than with deoxyviolacein (IC_50_ > 50 μM).

## Conclusions

We have utilized synthetic operons encoding enzymes for complete or partial pathways for the biosynthesis of violacein or deoxyviolacein in *E. coli* strains, and investigated the biological activity of the produsts. Deoxyviolacein, in spite of its lower antiparasitic activity, might be a better starting point than violacein for the development of a novel antiparasitic drug due to its low toxicity to human cells. However, it is important to note that we did not investigate the import of either compound by the target cells, and so cannot exclude the possibility that deoxyviolacein’s lower toxicity was due to an inefficient import into mammalian or parasite cells.

## Additional files

Additional file 1: **Figure S1.** Chemical structure (www.chemspider.com) and ^1^H spectra of Violacein and Deoxyviolacein. Chemical structure (www.chemspider.com) and ^1^H spectra of Violacein and Deoxyviolacein. (PDF 216 kb)

Additional file 2: **Figure S2.** Cytotoxicity of deoxyviolacein and violacein to HepG2 cells. Morphological changes of HepG2 cells treated with 0.8 to 100 uM of deoxyviolacein and violacein. **Figure S3.** Cytotoxicity of deoxyviolacein and violacein to CHO-745 cells. Morphological changes of CHO-745 cells treated with 0.8 to 100 uM of deoxyviolacein and violacein. (PDF 4205 kb)
