# Supplementary figures and images for: Antiplasmodial and trypanocidal activity of violacein and deoxyviolacein produced from synthetic operons
Source: BMC Biotechnol. 2018 Apr 11;18:22. doi: 10.1186/s12896-018-0428-z (PMC5896143; doi:10.1186/s12896-018-0428-z)

# Violacein

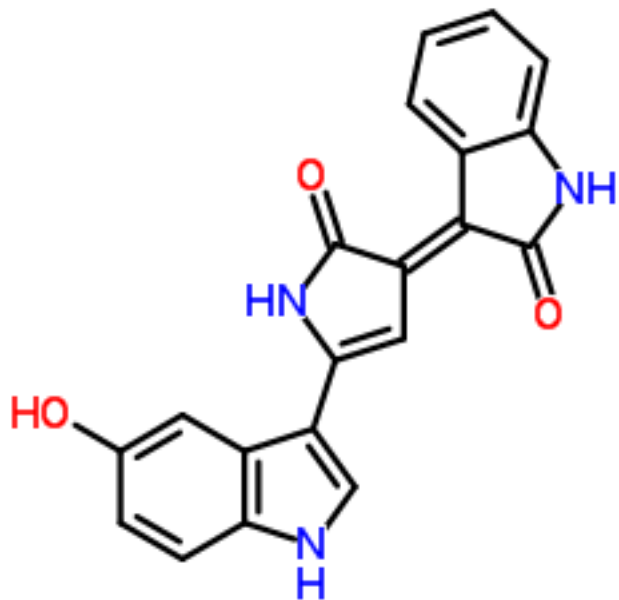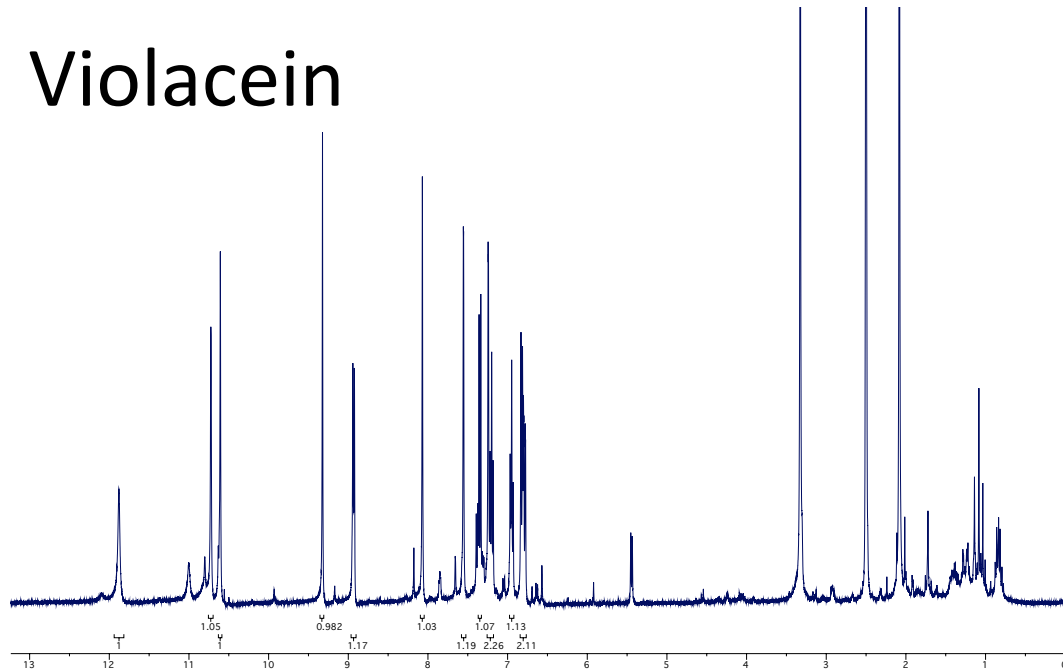

# Deoxyviolacein

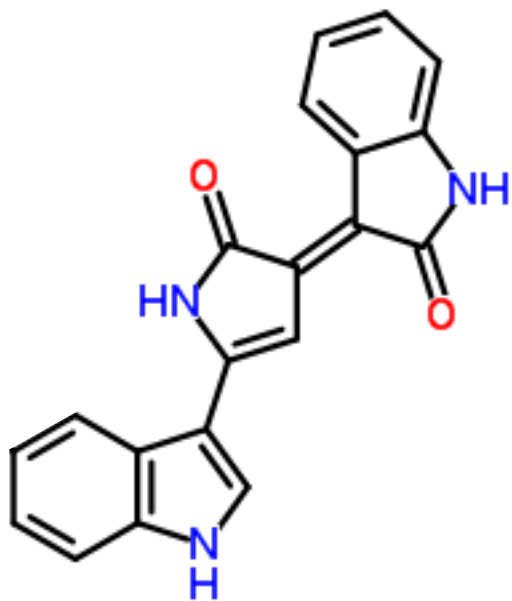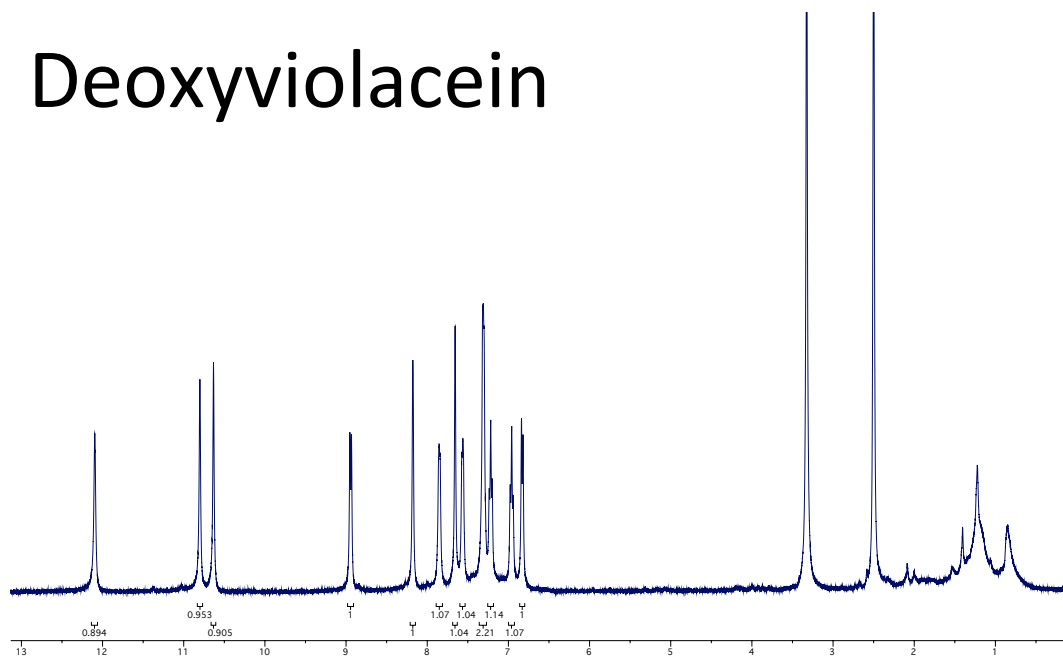

Supplement: Supplementary file 1 — Figure S1. Chemical structure (www.chemspider.com) and 1H spectra of Violacein and Deoxyviolacein. Chemical structure (www.chemspider.com) and 1H spectra of Violacein and Deoxyviolacein. (PDF 216 kb) [file 12896_2018_428_MOESM1_ESM.pdf]

# HepG2 cells

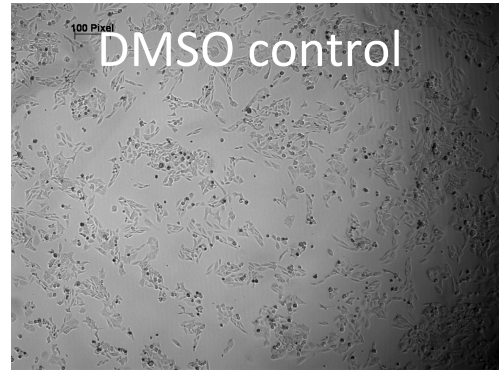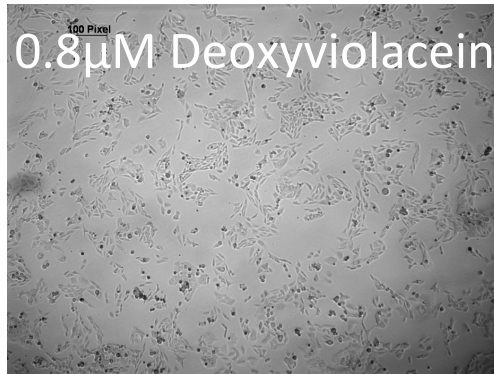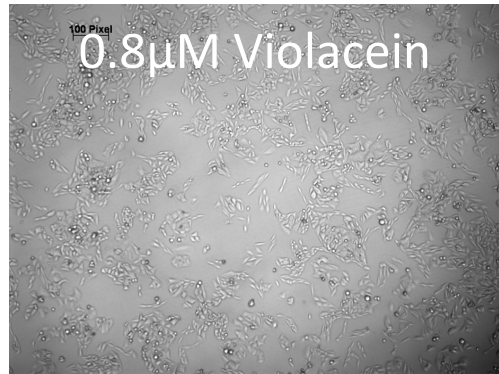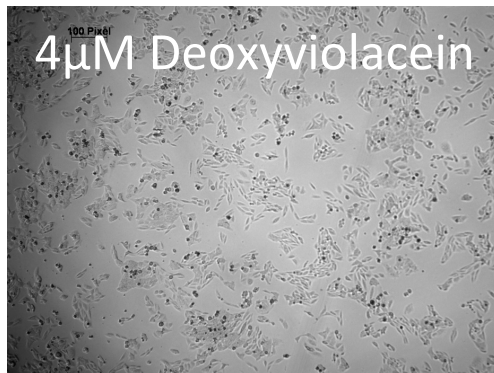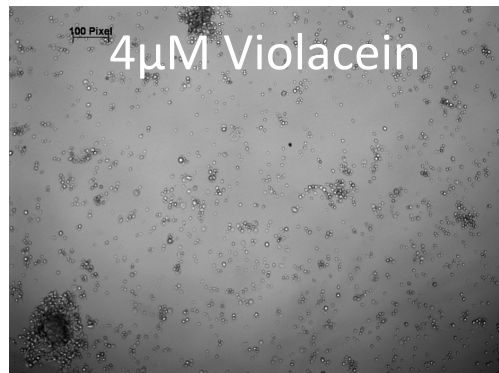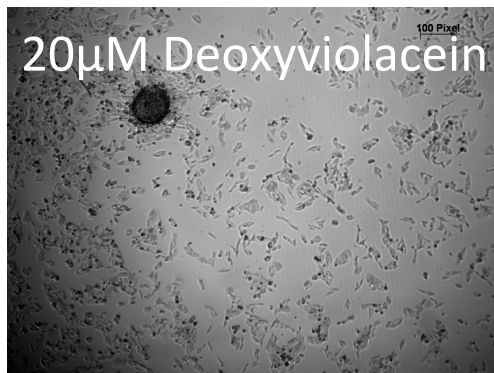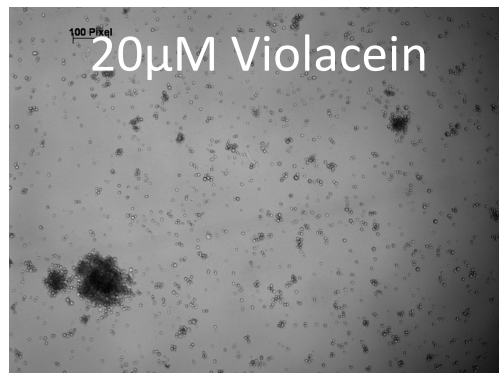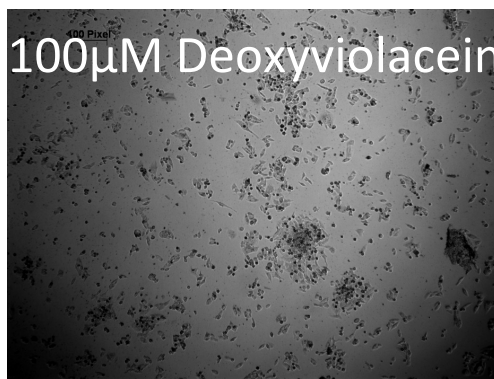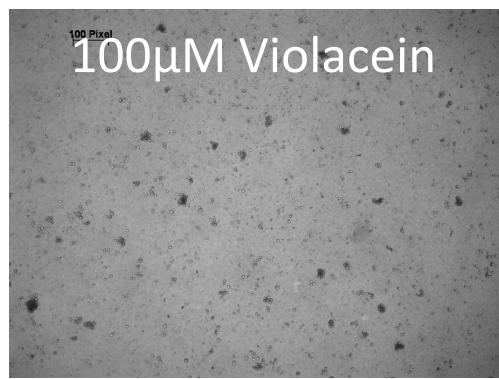

# CHO-745

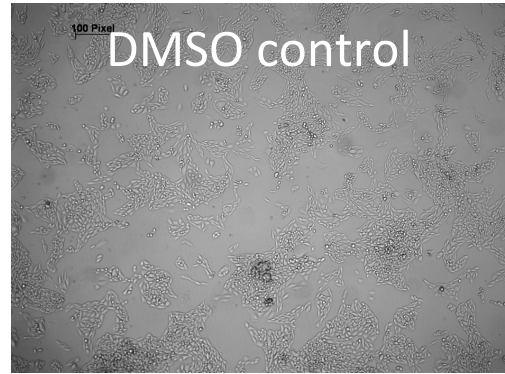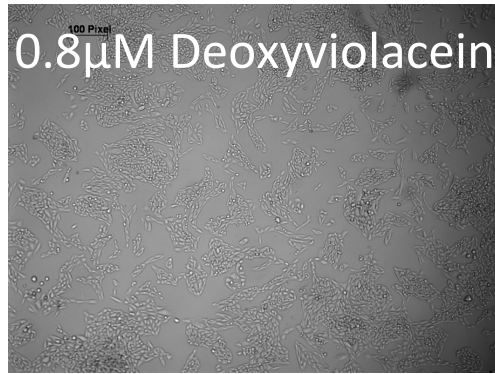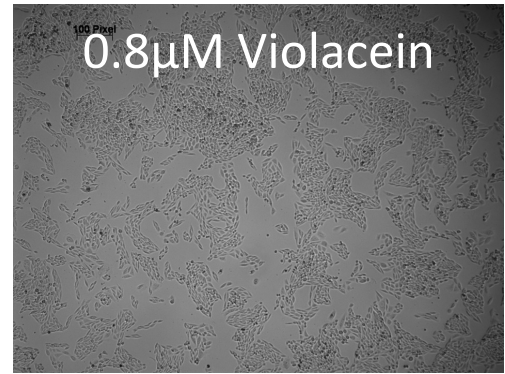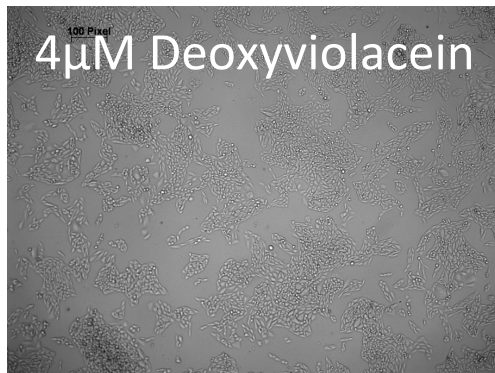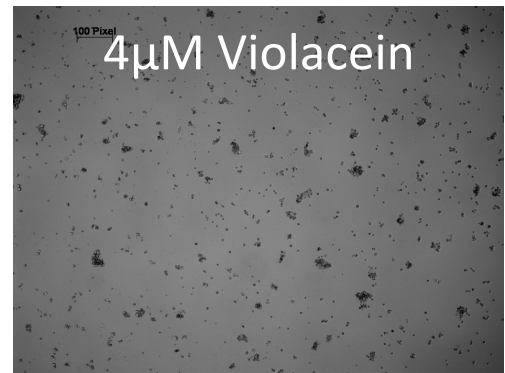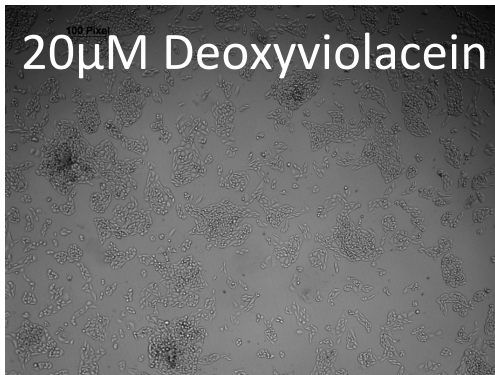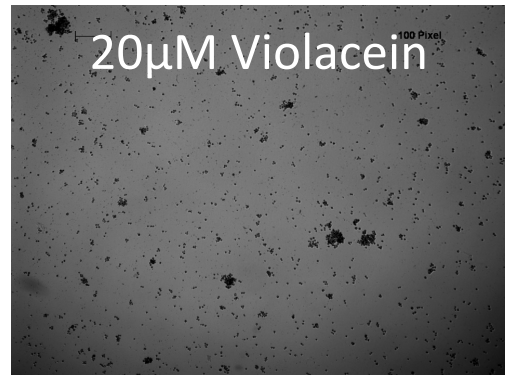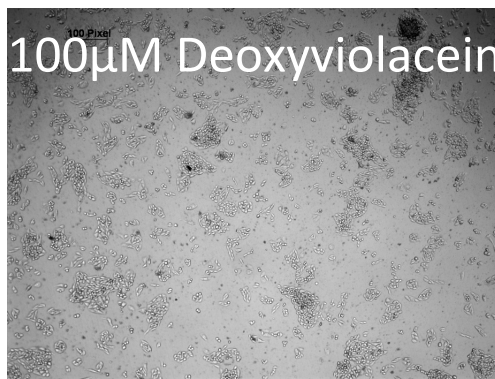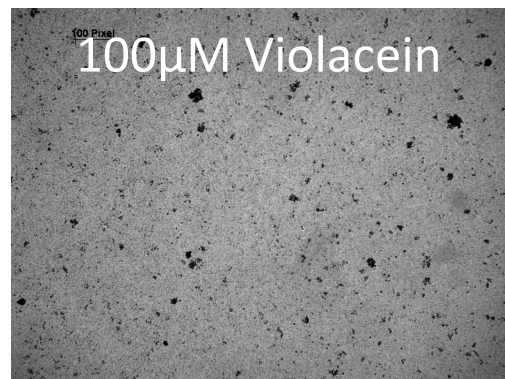

Supplement: Supplementary file 2 — Figure S2. Cytotoxicity of deoxyviolacein and violacein to HepG2 cells. Morphological changes of HepG2 cells treated with 0.8 to 100 uM of deoxyviolacein and violacein. Figure S3. Cytotoxicity of deoxyviolacein and violacein to CHO-745 cells. Morphological changes of CHO-745 cells treated with 0.8 to 100 uM of deoxyviolacein and violacein. (PDF 4205 kb) [file 12896_2018_428_MOESM2_ESM.pdf]
